# Growth and fatty acid distribution over lipid classes in *Nannochloropsis oceanica* acclimated to different temperatures

**DOI:** 10.3389/fpls.2023.1078998

**Published:** 2023-02-10

**Authors:** Narcís Ferrer-Ledo, Lars Stegemüller, Marcel Janssen, René H. Wijffels, Maria J. Barbosa

**Affiliations:** ^1^ Bioprocess Engineering, Wageningen University and Research, Wageningen, Netherlands; ^2^ Faculty of Biosciences and Aquaculture, Nord University, Bodø, Norway

**Keywords:** microalgae, *Nannochloropsis oceanica*, temperature stress, lipid classes, eicosapentaenoic acid

## Abstract

After light, temperature is the most relevant environmental parameter in outdoors cultivation of microalgae. Suboptimal and supraoptimal temperatures negatively impact growth and photosynthetic performance with a subsequent effect on lipid accumulation. It is generally recognised that lower temperatures trigger an increase in fatty acid desaturation while higher temperatures trigger the opposite reaction. The effect of temperature on lipid classes has been less studied in microalgae and in certain cases, the effect of light cannot be completely excluded. In this research, the effect of temperature on growth, photosynthesis, and lipid class accumulation in *Nannochloropsis oceanica* was studied at a fixed light gradient with a constant incident light intensity (670 μmol m^-2^ s^-1^). A turbidostat approach was used to achieve temperature acclimated cultures of *Nannochloropsis oceanica*. Optimal growth was found at 25-29°C, while growth was completely arrested at temperatures higher than 31°C and lower than 9°C. Acclimation to low temperatures triggered a decrease in absorption cross section and photosynthesis rates with a tipping point at 17°C. Reduced light absorption was correlated with a decrease in content of the plastid lipids monogalactosyldiacylglycerol and sulfoquinovosyldiacylglycerol. The increase of diacylglyceryltrimethylhomo-serine content at lower temperatures indicated a relevant role of this lipid class in temperature tolerance. Triacylglycerol content increased at 17°C and decreased at 9°C emphasising a metabolic switch in stress response. Total and polar eicosapentaenoic acid content remained constant at 3.5 and 2.4% w/w, despite the fluctuating lipid contents. Results show an extensive mobilisation of eicosapentaenoic acid between polar lipids classes at 9°C to ensure cell survival under critical conditions.

## Graphical Abstract

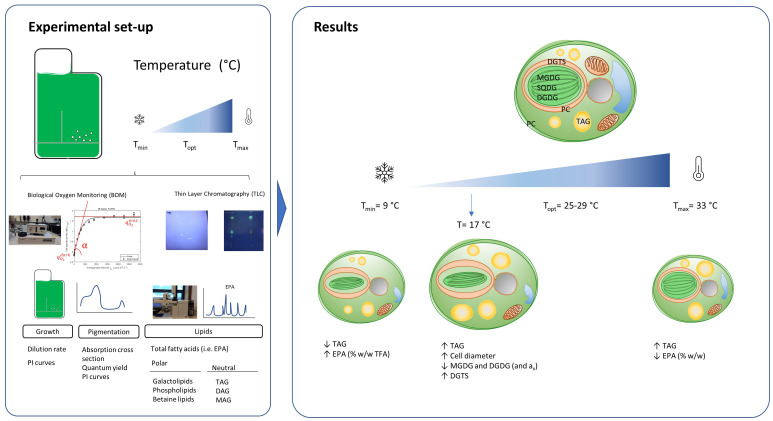

## Highlights

- A tipping point response was observed at the temperature of 17°C with accentuated changes in growth, photosynthesis and lipid composition.
- The increase in fatty acid content and EPA in the betaine lipid diacylglyceryltrimethylhomo-serine features an important role of this lipid class in low temperature adaptation.
- EPA content remained constant at 3.5% w/w at low temperatures, increasing only its percentage in lipids.

## 1 Introduction

Eicosapentaenoic acid (EPA) and docosahexaenoic acid (DHA) are two classes of polyunsaturated fatty acids (PUFA) which are classified as omega-3 fatty acids. Omega-3 fatty acids (ω-3 FA), in combination with omega-6 fatty acids, play an important role in human health by modulating inflammatory processes. Epidemiological (Dyerberg, 1989) and intervention studies (Burr et al., 1989; Yokoyama et al., 2007) indicated that diets including ω-3 FA could potentially reduce mortality rates associated with cardiovascular disorders. Based on that, but also on gender and age, an intake of 250-500 mg per day of DHA and EPA is recommended by health authorities for the prevention of chronic cardiovascular diseases (Gebauer et al., 2006; EFSA, 2010). Fish and oil supplements are currently the main sources of ω-3 FAs for human and animal consumption; the oil supplements being mainly derived from fish and krill (Adarme-Vega et al., 2012; Saini et al., 2021). The uncontrolled use of krill and fish stocks from the environment has nhegative implications for the species biodiversity and food security (FAO, 2020; Jenkins et al., 2009). In addition, the aquaculture industry largely increased in the last decades to meet the increasing demand for fish and, as such, limit wild fish captures. Up to today, the outcome of this young industry is still insufficient to meet the global demands for fish. The sector still relies on small edible fish to feed and grow the catalogue of farmed fishes, resulting in extra pressure for wild fish catches (Oliver et al., 2020). Overall, there is a need for alternative sources of ω-3 FA which can overcome the environmental footprint of the current sources.

Microalgae represent a promising feedstock of biochemical compounds for the fuel, food, feed, cosmetic, pharmaceutical and commodity sectors (Draaisma et al., 2013; Enzing et al., 2014; Acién Fernández et al., 2021). From an ecological point of view, marine algae species are found at the beginning of trophic chains due to their innate ability to synthesise and accumulate ω-3 FAs. *Nannochloropsis oceanica*, a marine species from the Eustigmatophyceae class, is an oleaginous microalgae species of commercial interest due to its high content of EPA found under nutrient replete conditions (2-4% w/w, 20-30% lipid basis; Sukenik, 1991). Members of the *Nannochloropsis* genus have been successfully cultivated in outdoors conditions (Chini Zittelli et al., 2003; de Vree et al., 2016; Carneiro et al., 2020), they can inhabit different environments (freshwater, marine and brackish waters), they can accumulate lipids up to 60% of their DW (Benvenuti et al., 2016; Janssen et al., 2018), and genomic and bioinformatic tools for this strain are under continuous development (Naduthodi et al., 2018; Gong et al., 2020). All these characteristics make this oleaginous model organism a promising single-cell oil factory and unique EPA feedstock (Al-hoqani et al., 2017).

Microalgal oils can be classified into polar and neutral lipids, and their relative cellular composition will determine the quality of the microalgae as well as their final application. For instance, oil rich in triacylglycerols could be of use for fuel applications while oil rich in phospholipids (or glycolipids) could be of use for cosmetic, nutraceutical or pharmaceutical applications (Lordan et al., 2017; Traversier et al., 2018). *N. oceanica* produces triacylglycerols (TAG) and diacylglycerols (DAG) as neutral lipids, while glycolipids, phospholipids and betaine lipids are the main polar lipids reported (Sukenik et al., 1989). The glycolipids monogalactosyldiacylglycerol (MGDG), digalactolsyldiacylglycerol (DGDG) and sulfoquinovosyldiacylglycerol (SQDG) are mainly present in the thylakoid membranes of the chloroplast and their function is linked to photosynthesis. Phospholipids comprise phosphatidylcholine (PC), phosphatidylethanolamine (PE), phosphatidylglycerol (PG) and phosphatidylinositol (PI) which, besides a structural role, many of them are also essential for the organisation of the photosystems I and II. Lastly, betaine lipids are a unique class of lipids present in some eukaryotes such as fungi, protozoa, bacteria and microalgae. Diacylglyceroltrimethylhomo-serine (DGTS) is the most abundant betaine lipid present in *N. oceanica* and it plays an active role in adaptation to low temperature (Murakami et al., 2018) and phosphate starvation (Mühlroth et al., 2017).

EPA is located either in the neutral or the polar lipid fraction, and the distribution of EPA over polar and neutral lipids might influence the food quality of microalgae-derived oils. It was shown that EPA esterified in polar lipids is more efficiently incorporated into membranes than EPA esterified in neutral lipids (Lordan et al., 2017). Previous studies showed that between 40 and 50% of EPA in *Nannochloropsis* species is accumulated in the galactolipids MGDG and DGDG under nutrient-replete conditions. Under nutrient-deplete conditions such as nitrogen starvation, EPA was mainly accumulated in TAG suggesting a role as a PUFA depot. Besides the presence in galactolipids and TAG, EPA is also present in DGTS, and to a lesser degree, in the phospholipids PG, PC and PE. While a biosynthetic role has been associated with phospholipipds such as PE, the role is less known in DGTS. Some studies hypothesised a role as a polar depot of PUFAs under certain stress conditions, and other studies hypothesised a certain involvement in biosynthesis.

In outdoor conditions, light is the most limiting parameter for growth and lipid synthesis. After light, temperature is the most relevant cultivation parameter since it is seldomly controlled. The lack of control could result in temperature fluctuations of 10 °C between maximum and minimum with negative effects on both biomass and lipid productivities (Béchet et al., 2010). On one side, low temperatures exert a (1) reduction of enzymatic activities related to among others carbon and nitrogen assimilation, (2) rigidification of cell and organelle membranes, and (3) reduction of the electron transfer chain. On the other side, high temperatures lead to opposite effects such as, (1) denaturation or inactivation of enzymes, (2) increase of membrane fluidity, or (3) changes in the cellular osmolarity levels (Barati et al., 2019; Hüner et al., 2022). These metabolic changes ultimately generate an imbalance in the cellular redox state which is compensated by changes in the regulation of the electron, energy and carbon partitioning. While the effect of suboptimal temperatures on electron and energy regulation has been widely studied (Hüner et al., 2022), less is known about possible changes in carbon partitioning, and especially polar lipid class remodelling (Morales et al., 2021). Triacylglycerols are well-known sinks for carbon, electrons and energy under these conditions and they are hypothesised to serve as a depot of PUFAs (Solovchenko, 2012). Concerning polar lipids, microalgae respond to low temperatures by increasing the carbon number and desaturation of fatty acids to improve membrane fluidity (Thompson, 1996; Harwood, 1998). It is, however, less studied how polar lipid classes change at different temperatures.

The effect of temperature on growth and FA composition has been extensively studied in *Nannochloropsis* species (**Table 1**). Several studies demonstrated that EPA content increased at low temperatures in *Nannochloropsis* species (Hoffmann et al., 2010; Wei et al., 2015; Sá et al., 2020), and other microalgae such as *Rhodomonas sp* (Oostlander et al., 2020). Low temperatures favour the activation of specific desaturases and therefore, the increased synthesis of PUFAs (Harwood, 1998). In most of these studies, the effect of temperature was studied in batch, thus evaluating the short-term response of the culture. Besides, under batch conditions approach, the effect of temperature is not assessed indistinctively from the influence of light since biomass growth leads to a daily decrease in the average light intensity of the culture. It is important therefore to learn how growth and lipid synthesis in light acclimated cultures are affected by different temperatures. While most previous studies were done in a range of incident light intensities between 60 and 250 μmol m^-2^ s^-1^, fewer studies have been done at light regimes close to saturating light intensities resembling outdoor conditions.

**Table 1:** Overview of studies evaluating the temperature effect on lipids from *Nannochloropsis* species.

Strain	Cultivation mode	Parameters	Lipids	Source
*N. salina*	Turbidostat	Temperature (17, 21, 26°C) and Nitrate (1800, 600, 300, 150, 75 μmol L^-1^)	TFA, EPA	(Hoffmann et al., 2010)
*Nannochlorospsis* sp.	Batch	Light, nitrogen and Temperature (14, 19, 24, 28 and 32°C)	EPA	(Sukenik et al., 1993)
*N. oceanica*	Batch	Light, nitrogen and Temperature (15, 20, 25, 30°C)	EPA	(Sá et al., 2020)
*N. oceanica*	Batch	Light and Temperature (from 13 to 32°C)	Not measured	(Sandnes et al., 2005)
*N. salina*	Batch	Light and Temperature (from 13 to 32°C)	TFA, EPA	(Van Wagenen et al., 2012)
*N. salina*	Batch	Temperature (5, 10, 15 and 20°C)	TFA, EPA, Lipidomics.	(Willette et al., 2018)
*N. oculata*	Batch	Temperature (20, 25, 30 and 35°C)	TFA, EPA and polarity	(Wei et al., 2015)
*Nannochloropsis sp FIKU036*	Batch	Temperature (25, 30, 35°C)	TFA, EPA	(Chaisutyakorn et al., 2018)
*Nannochloropsis oculata CS-179*	Batch	Temperature (10, 15, 20, 25 and 30°C)	Only EPA composition	(Ma et al., 2011)
*Nannochloropsis salina CCMP1776*	Batch	Temperature (5, 10, 15, 25°C)	Lipidomics	(Gill et al., 2018)
*N. salina and N. oculata*	Batch	Temperature (8, 14, 20, 26°C)	FA and pigments	(Aussant et al., 2018)

In this research, we investigated how temperature affects the distribution of fatty acids between lipid classes, with special attention to EPA and polar lipids, in long-term light acclimated cultures of *N. oceanica*. To achieve this goal, the effect of temperature ranging from critical, suboptimal, to supraoptimal was studied on steady-state cultures of *N. oceanica*. A turbidostat operation mode was used to maintain an equal light gradient over the reactor depth for all different temperature conditions. Growth, acclimation and photosynthesis performance were evaluated at each temperature to assess the long-term response. Photosynthesis-Irradiance curves were developed using oxygen evolution at different incident light conditions, and parameter estimation was applied to further investigate the effect on photosynthesis. Total fatty acids were analysed at each temperature while fractionation of the fatty acids between the different lipid classes was only done for representative sub-, supra- and optimal conditions.

## 2 Materials and methods

### 2.1 Strain, medium and pre-cultivation conditions

*Nannochloropsis oceanica*, kindly provided by NECTON S.A. (Olhão, Portugal), was pre-cultured in sterile 250 mL Erlenmeyer flasks with 100 mL of filter-sterilised (pore size of 0.2 µm; Sartorius, Germany) medium. Medium composition was based on artificial seawater and micronutrients adapted from the recipe of NutriBloom plus (Phytobloom, Olhão, Portugal) resulting in: NaCl 419.2 mM; MgCl_2_·6H_2_O 48.2 mM; NaNO_3_ 35.3 mM; Na_2_SO_4_ 22.5 mM; CaCl_2_·2H_2_O 5.4 mM; K_2_SO_4_ 4.9 mM; KH_2_PO_4_ 0.73 mM; Na_2_EDTA·2H_2_O 52.8 μM; FeCl_3_·6H_2_O 40 μM ZnSO_4_·7H_2_O 4 μM; MgSO_4_·7H_2_O 4 μM; MnCl_2_·4H_2_O 2 μM; Na_2_MoO_4_·2H_2_O 0.2 μM; CoCl_2_·6H_2_O 0.2 μM; CuSO_4_·5H_2_O 0.2 μM. Maintenance cultures were cultivated under orbital agitation, at 25°C, low incident light of 20-50 µmol m^-2^ s^-1^ with 16:8 L:D cycles. Pre-cultures for reactor cultivation were grown in an orbital shaker HT Multitron Pro (Infors HT, Switzerland) at 120 rpms, 25°C, 100-120 µmol m^-2^ s^-1^ (warm-white LED) in 16:8 L:D cycles and 0.2% CO_2_ enriched headspace. All cultures were maintained at pH=7.5 by the addition of HEPES at a final concentration of 20 mM.

Medium composition of the reactor was the same as for the cultures with the exception that reactor medium did not contain HEPES. Additionally, NaNO_3_ and KH_2_PO_4_ concentrations were increased to 58.8 and 2.76 mM, respectively, to avoid nutrient limitations. Final pH medium was adjusted to 6 to avoid nutrient precipitation during reactor operation.

### 2.2 Photobioreactor operation mode and set-up

Pre-cultures were inoculated to heat-sterilised airlift-loop flat panel Labfors 5 photobioreactors (Infors HT, Switzerland). The reactor has an illuminated surface area of 0.08 m^2^, a depth of 20.7 mm and a working volume of 1.8 L. Incident light was provided with a set of 260 water-cooled high-power LED lights (28 V, 2.3 Watt per LED) at 670 μmol.m^-2^.s^-1^ with a photoperiod of 16h of constant light and 8 hours of darkness. pH was measured and controlled at 7.5 ± 0.05 by acid (0.9 M, H_2_SO_4_) or base (1 M, NaOH) addition. Mixing was done by sparging air at 0.56 vvm enriched with 2% CO_2_. Evaporation losses were minimised with a condenser located on top of the reactor. Temperature was measured and controlled with a water jacket directly connected to a cryostat. In this research, the following temperature setpoints were studied: 9, 13, 17, 21, 25, 29, 31 and 33°C.

Reactors were operated continuously in turbidostat mode. Light on the rear of the reactor was measured by a secondary light sensor (LI-190SA 2ϖ quantum sensor, Li-Cor, USA) and controlled at 15 μmol m^-2^ s^-1^, above the experimental compensation light intensity (I_ph,c_) of 4 μmol m^-2^ s^-1^ estimated from Eq 1:


(1) $$I_{ph,c}=\;\frac{q_{ph}^{m}}{a_{x}}$$

where $q_{ph}^{m}$ is the specific photon supply rate for maintenance purposes assumed to be 3.53 mmol_ph_ gDW^-1^ h^-1^ for *Nannochloropsis sp* (Barten et al., 2022) and a_x_ the averaged absorption cross-section assumed to be 0.27 m^2^ gDW^-1^ at low light conditions (Janssen et al., 2018). Cultures were first grown in batch at an initial OD_750_ of 0.15-0.2 and incident light of 200 μmol m^-2^ s^-1^. The incident light was increased stepwise with the growth of the culture, first to 400 μmol m^-2^ s^-1^, and then to 670 μmol m^-2^ s^-1^. At this light intensity, turbidostat mode started and filter-sterilised medium was added automatically whenever the light intensity at the rear dropped below the setpoint. The overflow (V_Harvest_) was pumped out to an empty vessel and it was collected and measured every day two hours after the beginning of the light period. The dry weight (DW) biomass concentration and the absorption cross section (a_x_) were measured to assess the physiology and stability of the culture. It was considered that the cultures reached the steady-state when the dilution rate, DW and a_x_ did not change more than 10% for at least 3 residence times. At steady state, samples for the measurement of DW, a_x_, cell concentration and oxygen evolution were directly taken from the reactor. The experiment at 9°C was not sampled at steady-state, but once the DW and the a_x_ did not change more than 10%. The V_Harvest_ line was connected to an empty vessel in order to collect biomass for biochemical analysis. Collection was done for 4 hours under dark conditions and the vessel was placed on ice. The V_Harvest_ collected was measured, centrifuged (5 min, 4200 g) and washed twice with ammonium formate (0.5 M) in order to remove any traces of salts. The supernatant was discarded and the pellet was flushed with N_2_ gas, sealed and stored at -20°C until further use.

After the collection of samples for one temperature, the experiment was concluded and the temperature setpoint was reduced or increased by a change of 2°C per day. After reaching the setpoint required, a new experiment started and the culture was monitored until a new steady state was achieved. Sampling at steady state was done on three different days. The experiment at 25°C was repeated in a different photobioreactor to assess the reproducibility of the results.

### 2.3 Offline analysis

Dry weight was measured by assessing the difference in weight of a known volume of culture on pre-weighted Whatman glass microfiber filters (55 mm; GE Healthcare, USA). Shortly, filters were pre-washed with deionised water, dried in the oven (100°C) overnight and cooled down in the desiccator for 2 hours and weighted. A culture volume containing 2-5 mg of biomass was filtered in pre-weighted filters and washed with ammonium formate (0.5 M) to remove the excess of salts from the biomass. Filters were dried for 24h, placed in a desiccator for at least 2h and weighted afterwards. Measurements were done in triplicate.

Cell concentration was measured in a Coulter Multisizer 3 (Beckman Coulter Inc, USA). Samples with an approximate optical density at 750 nm of 0.1-0.2 were diluted 100 times in Isoton reagent (ISOTON™ II Dilutant, Beckman Coulter Inc., USA) and measured with an aperture tube of 50 μm. Cell concentration and diameter were calculated from the number of particles distributed between 2 and 5 μm after analysing 1 mL of diluted sample.

The absorption spectrum of undiluted cultures was measured in a UV-2600/2700 Spectrophotometer (Shimadzu, Japan) equipped with an integrating sphere for light scattering correction. The absorbance from biomass scattering (740-750 nm) was substracted to the absorbance from 400 to 700 nm and normalised to the DW of the sample and light path (2 mm). The averaged-specific absorption cross section was obtained after averaging the relative absorbance values from 400 to 700 nm.

The maximum quantum yield of PSII photochemistry (QY_max_) was measured in diluted dark-adapted samples with a fluorometer (AquaPen-C AP-C100, Photon Systems Instruments, Czech Republic). The maximal fluorescence signal (F_m_) and fluorescence baseline (F_o_) from diluted cultures (OD_750_ ~ 0.1-0.2) were obtained after incubation in dark conditions for 10-15 min. Maximal quantum yield of PSII was measured as follows:


(2) $$QY_{max}=\;\frac{F_{m}-F_{o}}{F_{m}}=\frac{F_{v}}{F_{m}}$$

Respiration and photosynthesis rates were measured by monitoring the oxygen evolution at different incident light intensities in an Oxytherm+P system (Hansatech Instruments Ltd, England). This is a liquid phase system with a cuvette of 9 mm diameter illuminated from one side with white LEDs. An O_2_ electrode was prepared according to manufacturer’s instructions. Prior to measurements, the electrode was calibrated with air (100% saturation) and N_2_ gas (0% saturation) with reactor medium adjusted to pH 7.5 and 40 mM final HEPES concentration. Na_2_S_2_O_4_ (1-3 mg) was added for the 0% saturation calibration to further remove dissolved oxygen. Calibration was done every time the device was used and according to the temperature in the reactor. Temperature was controlled during the whole measurement. Prior to the analysis, the culture was diluted to a final known biomass concentration of 0.15-0.2 g L^-1^ and dissolved O_2_ concentration was reduced to 25% of the saturation value. Sodium bicarbonate (NaHCO_3_) was added to a final concentration of 10.2 μM to prevent carbon limitation during the measurement. Oxygen evolution was measured according to the following irradiance setup plan: darkness (6 min), 10 (4), 20 (3), 40 (2, 80 (2), 160 (1), 250 (1), 350 (1), 500 (1), 750 (1), 1000 (1), 1500 (1), 2000 (1), 2500 (1), 3000 (1). Dissolved oxygen concentration (μmol_O2_ L^-1^) was recorded in intervals of one second and the volumetric oxygen rate (μmol_O2_ min^-1^ L^-1^) was calculated after stabilisation of the slope.

Total fatty acid quantification was done according to (Breuer et al., 2013), with the exception that no internal standard was added prior to disruption. Shortly, 5-10 mg of freeze-dried biomass were bead-beaten (3 cycles of 60 s at 2500 rpm with 120 s break) and transferred to a fresh new tube. Sonication (10 min, 80 kHz) and a saline solution of 50 mM and 1 M NaCl at pH 7.5 were added to the disrupted extract. 3 cycles of extraction with 1 mL of chloroform and transfer to a fresh tube were applied. Chloroform was evaporated with N_2_ gas and the lipid extract was sealed with parafilm and stored at -20°C until further use.

Lipid classes were separated and their FA composition was analysed as described by Gros and Jouhet, 2018, with some minor differences. Shortly, after dilution of lipid extract in 1 mL of chloroform, a 50 μL sample was methylated (1 h, 100°C) and the total fatty acids were quantified by GC-FID. The volume corresponding to 750 nmol of FA was loaded in pre-activated Thin Layer Chromatography (TLC) silica plates (105721, Supelco, Germany) and different plates were used to separate polar and neutral lipids, respectively. Migration and identification of lipid classes in TLC plates were done accordingly to Gros and Jouhet, 2018 with the difference that a solution of primuline 0.5% v/v in 8:2 acetone: deionised water was employed as a staining agent. After identification, the silica from the spots was scrapped and collected into separate glass tubes. The content of the tubes was methylated (1 h, 100°C) and quantified by GC-FID by using the FAME standard pentadecanoic acid (CAS 1002-84-2, Merck, Germany). FA quantification was based on the relative response of a standard mix of fatty acid methyl esters (FAMEs) and normalised to the initial weighted DW. The FA content for each lipid class was normalised to the sum of all the FAs to estimate the FA composition per lipid class (% mol mol^-1^). The lipid class composition was based on the total content of FA found in each lipid spot normalised to the sum of all the different lipid and the number of acyl chains per lipid class. The content of FA in lipid classes (w/w) was calculated based on the FA composition, the lipid class composition and the TFAs.

### 2.4 Calculations

The dilution rate (D, d^-1^) of the turbidostats was calculated from the harvested overflow as follows:


(3) $$D=\;\frac{V_{Harvest}}{V_{r}}$$

where V_Harvest_ was collected after 1 day and V_r_ stands for the working volume of the reactor.

The volumetric absorption capacity was calculated as the product of the a_x_ and DW.

Photosynthesis-Irradiance (PI) curves were obtained from plotting the specific oxygen production rate (*q*_*O*_2__ ) to the incident light intensity (I_ph,o_). Volumetric oxygen production rates, measured from biological oxygen monitoring experiments, were normalised to the estimated DW in the light chamber.

The measured specific oxygen production rate is the result of the cellular photosynthesis and respiration rates (Eq. 4). Photosynthesis rates can be described as a function of the incident light intensities with the Jassby and Platt model (Jassby and Platt, 1976) as follows:


(4) $$q_{O_{2}}=\;q_{O_{2}}^{phot}-q_{O_{2}}^{dark}=q_{O_{2}}^{max}\tanh\left(\frac{\alpha \;I_{ph,o}}{q_{O_{2}}^{max}}\right)\;-q_{O_{2}}^{dark}$$

(5) $$\alpha ={\text{Y}}_{\text{oph}}{\text{ a}}_{\text{x}}$$

where $q_{O_{2}}^{dark}$ is the specific oxygen rate associated with respiration processes, $q_{O_{2}}^{max}$ is the maximal specific oxygen production rate, *α* is the slope of the linear phase at light limiting conditions and Y_oph_ is the yield of oxygen on light. Fitting the specific oxygen production rates as a function of the incident light intensity of each experiment to Eq. 4 resulted in an estimation of $q_{O_{2}}^{max}$ , *α* and $q_{O_{2}}^{dark}$ for each temperature evaluated.

### 2.5 Statistical analysis

Descriptive statistics were used to estimate the average and standard deviations of replicate measurements. The *post hoc* Tukey’s range test was done to assess changes in lipid classes and FA composition per lipid class at different temperatures with a confidence level of 95%. Matlab (MATLAB, 2010) was used for parameter estimation and data visualisation. Error propagation was used in case multiple arithmetic operations were required. R (R Core Team, 2021) was used for statistical analysis.

## 3 Results and discussion

### 3.1 Effect of temperature on growth and acclimation in continuous cultures of *Nannochloropsis oceanica*

**Figure 1:**
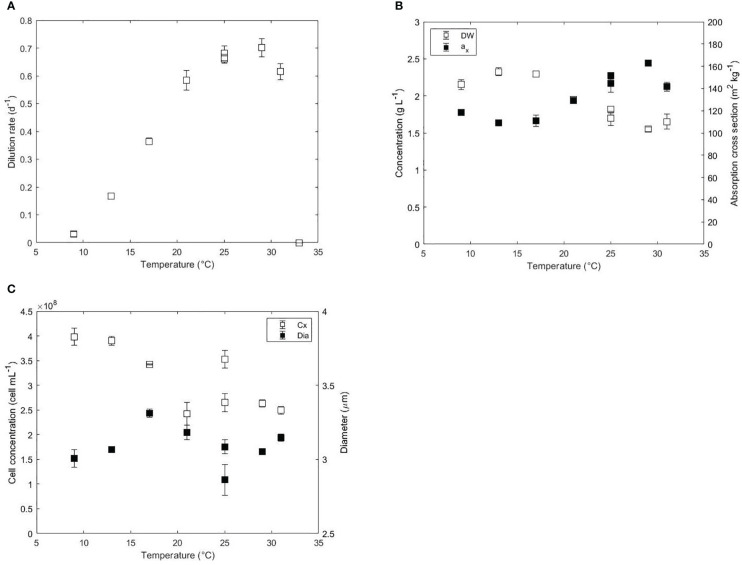
**(A)** Dilution rate (d^-1^), **(B)** biomass dry weight (g L^-1^), absorption cross section (m^2^ kg^-1^), **(C)** cell concentration (cell mL^-1^) and diameter (μm) as a function of the temperature (°C) in acclimated cultures of *N. oceanica*. Error bars represent the standard deviation of three measurements. Two independent runs were performed at 25°C to assess the reproducibility of the results.

Temperature is the parameter that exerts the most notorious effect on growth during microalgae cultivation after light. The effect of temperature on growth has been widely studied in *Nannochloropsis* species by several authors (**Table 1**). Except for the turbidostat studies of Hoffmann et al., 2010, most studies were done in batch and therefore light perceived by the cultures varied with culture growth. In many of those studies, a synergistic effect of temperature and light was studied without a distinction of the influence of each parameter. In this study, the light perceived by the culture was fixed for all experiments by keeping a constant light gradient over the depth of the reactor. In this way, the effect derived from temperature is the only variable evaluated. In continuous experiments, where light is the only limiting factor, the dilution rate measured is equivalent to the growth rate of the culture according to the cell mass balance over the reactor. The steady-state dilution rates at different temperatures are represented in **Figure 1A**. The highest dilution rate ranged between 0.68 and 0.70 d^-1^ and was found between 25 and 29°C. The dilution rate decreased sharply from 0.7 to 0 d^-1^ at supraoptimal values from 29 to 33°C, while the decrease was less steep at suboptimal temperatures from 0.68 d^-1^ at 25°C to 0.03 d^-1^ at 9°C. The abrupt drop in growth at supraoptimal temperatures indicates that this species is extremely sensitive to temperatures near 30°C. The optimal temperatures for growth found in this research agree with previous batch experiments with other *Nannochloropsis* strains. The optimal temperature for growth of *Nannochloropsis oceanica* was found between 26 and 28°C at different incident irradiances between 34 and 80 μmol m^-2^ s^-1^ (Sandnes et al., 2005). An optimal temperature for growth of 24-26°C was also found for *Nannochloropsis gaditana* (Van Wagenen et al., 2012), *Nannochloropsis sp* (Sukenik, 1991) and *Nannochloropsis oculata* (Wei et al., 2015).

In a turbidostat operation mode, where the outgoing light intensity is fixed, the light absorption area per volume of reactor is expected to be constant since the incident light intensity was also constant at daylight. Hence, the biomass dry weight (DW) and the averaged specific absorption cross section area (a_x_) of the biomass affect the volumetric light absorption capacity of the culture. The volumetric light absorption capacity was constant at 252 m^2^ L^-1^ for all temperatures studied except at 31°C, where it decreased to 233 m^2^ L^-1^ due to unknown reasons. Despite the constant light absorption, the DW and the a_x_ changed inversely both at low and high temperatures as shown in **Figure 1B**. At temperatures near optimal growth (25-29°C), biomass concentration and absorption cross section were 1.55-1.82 g L^-1^ and 145-163 m^2^ kg^-1^, respectively. From 29 to 17°C, the DW increased from 1.82 to 2.33 g L^-1^ and the a_x_ decreased from 163 to 109 m^2^ kg^-1^. The a_x_ increased at even lower temperatures from 109 m^2^ kg^-1^ at 17°C to 119 m^2^ kg^-1^ at 9°C. Data of absorbance spectra showed a higher absorbance between 420 and 480 nm, which indicates an increased content in carotenoids at 9°C (**Figure S1**). At temperatures higher than 29°C, the biomass concentration remained constant at 1.6 g L^-1^ while the a_x_ decreased from 163 to 142 m^2^ kg^-1^.

Cell concentration and cell diameter are presented in **Figure 1C**. Similar to the biomass DW, the cell concentration increased from 2.50E+8 cell mL^-1^ at 31°C to 3.98E+8 cell mL^-1^ at 9°C. Cell diameter was constant (~3.05 μm) at optimal and supraoptimal conditions but increased up to 3.31 μm at 17°C. At lower temperatures than 17°C, the diameter decreased to 3.00 μm. The decrease in diameter could explain the decrease of DW observed at 9°C. Previous short-term studies in *Chlorella*, *Scenedesmus* or *Mycrocistis* showed increased cell volumes and decreased chlorophyll contents at low temperatures (Maxwell et al., 1994; Staehr and Birkeland, 2006).

### 3.2 Effect of temperature on photosynthesis and respiration

**Figure 2:**
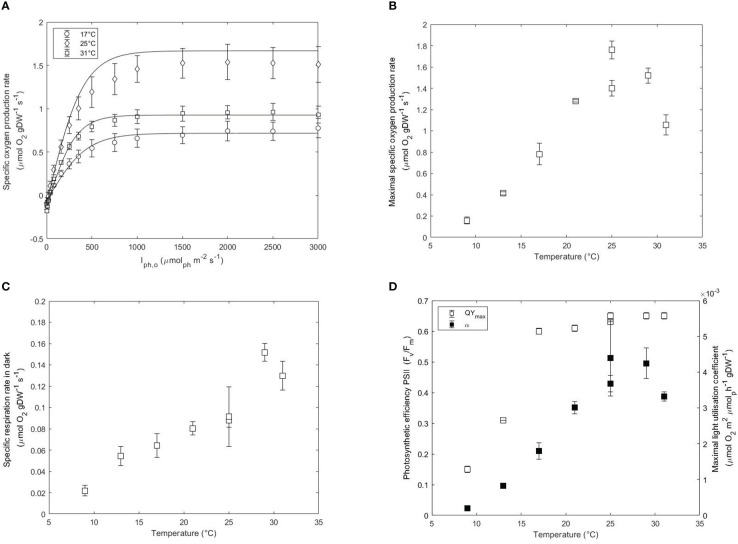
**(A)** Photosynthesis-Irradiance (PI) curves at 17, 25 and 31°C. Maximal specific oxygen production rate ( ${\text{q}}_{{\text{O}}_{2}}^{\text{max}}$ , μmol gDW^-1^ s^-1^) **(B)**, respiration rates in the dark ( ${\text{q}}_{{\text{O}}_{2}}^{\text{dark}}$ , μmol gDW^-1^ s^-1^) **(C)**, and QY_max_ (F_v_/F_m_) and maximal light utilisation rate (α, μmol O_2_ m^2^ μmol ph^-1^ gDW^-1^) **(D)** as a function of temperature in acclimated cultures of *N. oceanica*. Error bars represent the standard deviation of three measurements. Two independent runs were performed at 25°C to assess the reproducibility of the results.

The photosynthetic performance was evaluated by using oxygen evolution and chlorophyll fluorescence. Photosynthesis-Irradiance (PI) curves were developed for each temperature to gain understanding on the acclimation, maximal photosynthesis rates and respiration rates. After fitting the Jasby and Platt model to the specific oxygen production rates over incident light intensity, the maximal specific oxygen production rate ( $q_{O_{2}}^{max}$ ), the maximum light utilisation coefficient (α) and the specific oxygen respiration rate in the dark ( $q_{O_{2}}^{dark}$ ) were estimated. Examples of PI curves derived at 17, 25 and 31°C including their respective fitting results are shown in **Figure 2A**. The $q_{O_{2}}^{max}$ indicates the maximal photosynthetic capacity of the microalga and was strongly affected by low and high temperatures. In similarity to the dilution rate, the $q_{O_{2}}^{max}$ was maximal at 25°C (1.58 μmol_O2_ gDW^-1^ s^-1^) and decreased to 0.94 μmol_O2_ gDW^-1^ s^-1^ at 31°C and 0.14 μmol O_2_ gDW^-1^ s^-1^ at 9°C (**Figure 2B**). Low temperatures slow down the rates of repair of the photosynthetic apparatus and electron transport in the thylakoid membranes resulting in a redox imbalance (Mellis, 1999). As a result, fewer PSII reaction centers are functional at lower temperatures, which ultimately results in a decreased maximal photosynthesis rate (Sakshaug et al., 1997). A slower electron transfer rate coupled to reduced enzymatic activities leads to a reduced energy pool in the cell (ATP and NADPH). The limited availability of ATP and NADPH will in turn affect relevant pathways such as carbon fixation, which is then reflected on a decreasing $q_{O_{2}}^{max}$ .

The decreasing $q_{O_{2}}^{max}$ at higher temperatures was also explained mainly by limitations in the carbon fixation reactions. Despite the high thermolability of the photosystems at high temperatures, the activase activity of the Rubisco enzyme was recognised as the main bottleneck (Crafts-Brandner and Salvucci, 2000; Salvucci and Crafts-Brandner, 2004; Allakhverdiev et al., 2008). Higher temperatures were shown to reduce the specificity of the Rubisco towards CO_2_ over O_2_, leading to the accumulation of phosphoglycolate. Phosphoglycolate needs to be regenerated to glycerate in order to reuse the carbon in the Calvin cycle and this can only be done *via* photorespiration (Peterhansel et al., 2010). The regeneration of carbon costs energy in the form of ATP and reducing power, resulting in lower photosynthetic rates. The maximal specific photosynthetic rate decreased by 40% (**Figure 2B**) at 31°C while the dilution rate only decreased by 10% (**Figure 1A**). The divergence existing between the dilution rate and the photosynthetic rates at 31°C suggests the occurrence of photorespiration but also the activation of other mechanisms generating additional energy. Heat stress was shown to increase the activity of PSI, hence increasing the redox state of the PSI (Sharkey and Zhang, 2010). As a result, a cyclic flow of electrons occurs to maintain the proton gradient across the thylakoid membranes and generate additional energy in the form of ATP. The activation of electron cyclic flow at 31°C could explain the maintenance of cell growth rates near the optimal despite the decay in photosynthetic rates.

Dark respiration accounts for the sum of all the metabolic processes that consume oxygen independently of light. Respiration rates are usually assumed to be 10% of the photosynthesis rate and can change with environmental conditions (J.Geider and Osborne, 1989). The respiration rates followed a similar trend to the photosynthetic rates by increasing with temperature. The estimated $q_{O_{2}}^{dark}$ was maximal at 29°C (0.15 μmol gDW^-1^ s^-1^) and decreased to 0.13 μmol gDW^-1^ s^-1^ at 31°C and 0.02 μmol gDW^-1^ s^-1^ at 9°C (**Figure 2C**). The high respiration rates at optimal conditions rather than suboptimal critical conditions are indicative of an increased rate of catabolism of carbohydrate storage to support higher growth rates. The $q_{O_{2}}^{dark}:q_{O_{2}}^{max}$ increased at lower and higher temperatures than 25°C suggesting higher metabolic requirements at supra- and suboptimal conditions (**Figure S2A**).

The maximum light utilisation coefficient (α) or slope of the PI curve is indicative of the transition from light-limiting to saturating conditions. α increased from 3.6E-3 μmol_O2_ m^2^ μmol_ph_^-1^ gDW^-1^ at 31°C to 4.7E-3 μmol_O2_ m^2^ μmol_ph_^-1^ gDW^-1^ at 29°C and then decreased at lower temperatures than 25°C to 2.2E-4 μmol_O2_ m^2^ μmol_ph_^-1^ gDW^-1^ at 9°C (**Figure 2D**). The α correlates with the yield of oxygen released per mole of photon (Y_oph_), providing additional information on the metabolic state of the culture. The yield decreased from 0.026 mol_O2_ mol_ph_^-1^ at 29°C down to 0.002 mol_O2_ mol_ph_^-1^ at 9°C, further confirming a loss of photosynthetic efficiency at lower and higher temperatures than optimal (**Figure S2B**). At higher temperatures than 29°C, the loss was less apparent. These results confirm that photorespiration occurs at 31°C and that a big share of energy for growth is granted by a predominant occurrence of cyclic photophosphorilation. In general, the obtained yields were relatively low to the expected theoretical values (Sakshaug et al., 1997) and this is explained by the protocol used. First of all, the incident light intensity instead of the average light intensity were used in the x-axis of the PI curve to estimate the slope. This resulted in an understimation of the real value since the non-absorbed light is also taken in the calculations (Saroussi and Beer, 2007). Secondly, the resulting values are dependent on the mathematical expression used. While the estimation of the $q_{O_{2}}^{max}$ is unvariable to the model used, the slope is highly dependent on it. In our study, we used the Jasby and Platt model to describe the relationship between light irradiance and photosynthesis but the use of other models would yield slightly different results. This might complicate the comparison and interpretation of data between different studies.

The QY_max_ was also measured at each temperature and the results are plotted in **Figure 2D**. The QY_max_ was highest between 31 and 25°C (0.65-0.64) and slowly decreased from 0.64 at 21°C to 0.61 at 17°C and then reduced by 2-fold every 4°C down to 0.15 at 9°C. A low QY_max_ suggests a low photosynthetic performance due to the occurrence of photoinhibition. Photosynthetic organisms dissipate the excess of absorbed light (not used in photochemistry) *via* non-photochemical quenching (NPQ). Distinct mechanisms have been studied in eukaryotes which are categorised depending on the relaxation times. Higher energy state quenching (qE) occurs in the order of seconds while photoinhibition quenching (qI) or zeaxanthin-dependent quenching (qZ) occur at longer times (Hüner et al., 2022). The prolonged adaptation of the culture to sub- and supraoptimal temperatures favors the occurrence of quenching mechanisms of longer time scales such as qI and qZ. Previous studies showed that QY_max_ and α of *Chlorella* cultures growing at 27°C were unaffected after short exposure to 5°C indicating that low temperatures do not interfere with the excitation process (Maxwell et al., 1994). However, chronic exposure to low temperatures combined with high saturating light intensities results in oxidative stress which is accentuated at lower temperatures. The observed decrease of QY_max_ and α at low temperatures support the activation of non-photochemical quenching processes. Lowering the temperature from 25°C to 17°C resulted in a decrease in α while the QY_max_ remained relatively constant. This discrepancy between QY_max_ and α, where QY_max_ decreased by only 8% and α decreased by 57% indicates that different quenching mechanisms are activated This possibly indicated that non-photochemical quenching occurred *via* fast relaxation routes such as qE or other routes of electron transfer such as the cyclic electron flow for ATP generation (Sakshaug et al., 1997). At temperatures lower than 17°C, the additional decrease in QY_max_ indicated membrane photodamage in the thylakoids and therefore the activation of new quenching routes of longer time scales. qI is a quenching mechanism occurring under photoinhibitory conditions that induce the inactivation of photosynthesis. Moreover, decreasing the temperature of the culture results in cells experiencing conditions similar to high saturating light intensities. The xanthophyll cycle is a photoprotective mechanism present in *Nannochloropsis oceanica* which aims at dissipating the excess of absorbed light under high light conditions. In this cycle, the product of violaxanthin de-epoxidation (zeaxanthin) acts as a quencher dissipating the excess of aborbed energy. As shown in the appendix, the increased absorption observed at 500 nm at temperatures lower than 25°C indicates the activation of the xanthophyll cycle.

At temperatures higher than optimal, the QY_max_ did not change, in contrast to lower temperatures. The discrepancy between the chlorophyll fluorescence method and the oxygen evolution further evidences that processes involved in carbon fixation are more limiting than the ones involved in light reactions during photosynthesis at high temperatures. Studies in acclimated and non-acclimated wheat and cotton plants to different temperatures showed that PSII photosynthetic activity was more resistant than Calvin cycle activity (Law and Crafts-brandner, 1999). Besides an increase in photorespiration, long-term exposure to high temperatures was shown to promote the uncoupling of the reaction centres of the photosystems from the light-harvesting complexes, as well as the unstacking of grana in the thylakoids (Hemme et al., 2014). Structural changes in the photosynthetic machinery could explain the lower a_x_ (**Figure 1B**), which occurred without significant changes in the absorption spectrum (**Figure S1**). Previous studies in plants reported changes in the light scattering properties of the chloroplast due to the formation of plastoglobules (Zhang et al., 2010).

Overall, these results emphasize that a different photosynthetic response occurs at low and high temperatures characterised by different mechanisms. Additionally, *N. oceanica* growth at lower temperatures is highly correlated to its photosynthetic activity, while temperatures higher than the optimum result in an uncoupling between growth and photosynthetic activities.

### 3.3 Total fatty acid content and distribution between polar and neutral lipids

**Figure 3:**
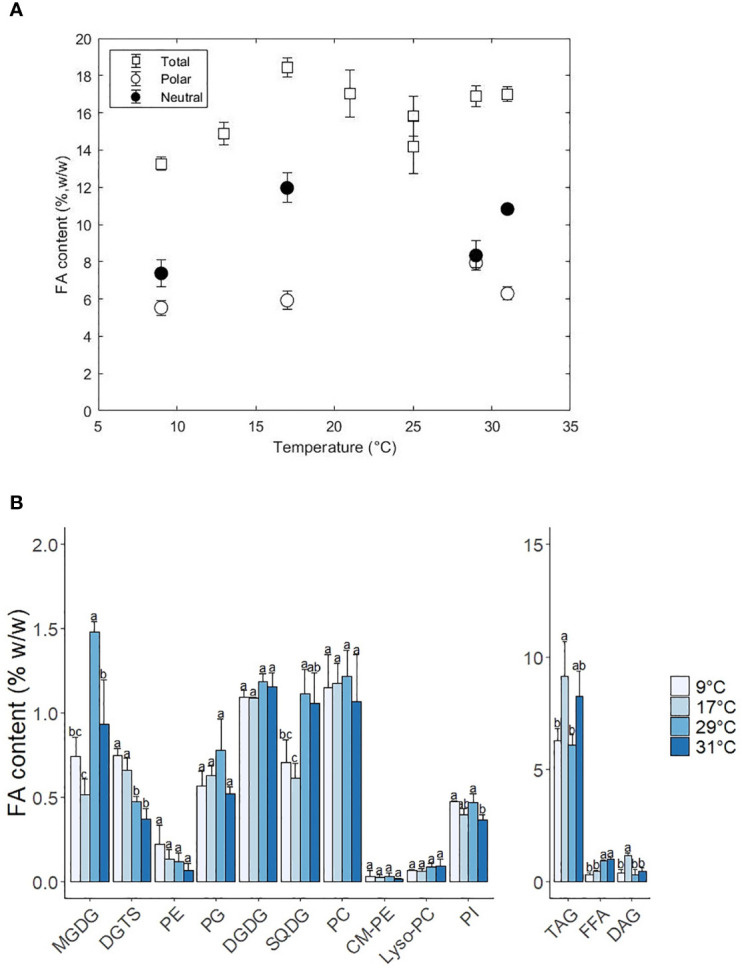
**(A)** Total fatty acid content (%, w/w) and distribution of fatty acids between polar and neutral fractions as a function of the temperature. **(B)** Change of lipid class content as a function of temperature. Standard errors indicate the standard deviation of 3 replicates. Letters a-c indicate statistically significant differences at P < 0.05 (Tukey test).

Besides, changes in photosynthesis rates, temperature variations affect intracellular lipid content and composition (Thompson, 1996; Solovchenko, 2012). **Figure 3A** depicts the changes in the content of total fatty acids (TFA) and the distribution between polar and neutral lipids for 4 selected temperature conditions. The temperature 29°C was representative of optimal growth, while temperatures 9 and 31°C were representative of suboptimal and supraoptimal conditions, respectively, at which growth was still present. Since temperature 17°C was observed as a tipping point in cell physiology and photosynthesis performance, lipids were also analysed and compared in the selected conditions. The TFA content remained constant at 17% w/w in the temperature range 17-31°C and decreased to 14.9% and 13.3% w/w at 13 and 9°C, respectively. Decrease in TFA was mainly due to a decrease in saturated fatty acids (SFAs), which decreased from 9.4% w/w at 31°C to 4.2% w/w at 9°C (**Table S1**). Polyunsaturated fatty acids (PUFAs) increased from 3.9% w/w at 31°C to a stable value of 4.8% w/w at 25°C and remained stable at lower temperatures. Fractionation of TFA between polar and neutral lipids showed that the polar lipid content was highest (7.9% w/w) near the optimal temperature (29°C) and decreased to 5.5% and 6.2% at 9°C and 31°C, respectively. Concerning the neutral lipids, the content at 29°C was 8.3% w/w, increased at lower temperature up to 12.0% w/w and decreased again down to 7.4% w/w due to the reduced photosynthesis rates. At supraoptimal conditions, the content increased up to 10.8% w/w. The decrease in neutral lipids correlated well with the reduced diameter observed at critical suboptimal temperatures.

### 3.4 FA distribution between lipid classes

**Figure 4:**
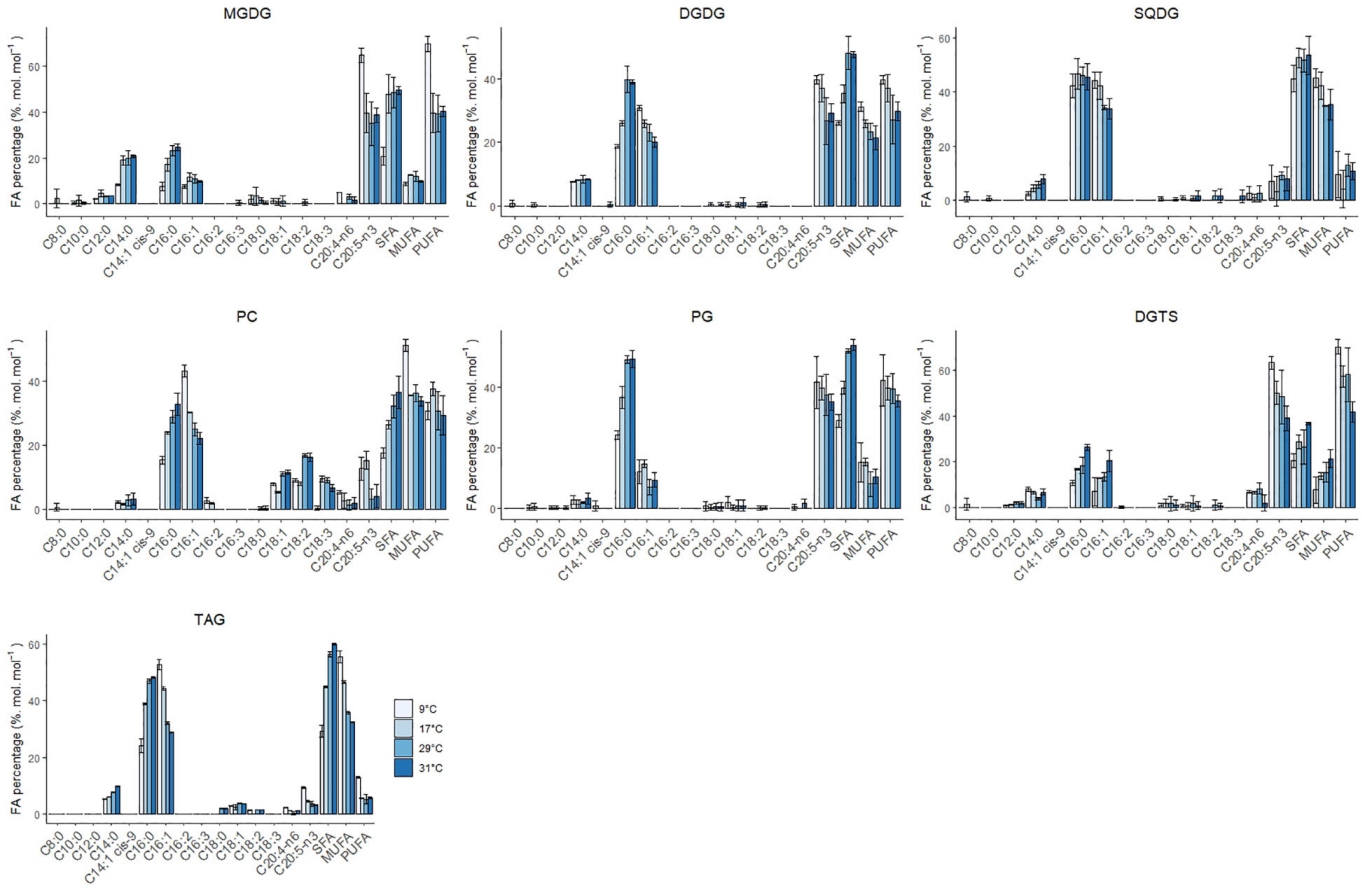
Individual FA, SFA, MUFA and PUFA composition of the lipid classes MGDG, DGDG, SQDG, PC, PG, DGTS and TAG at 9, 17, 29 and 31°C. Error bars indicate the standard deviation of three replicates.

Polar and neutral lipids were further separated into lipid classes with thin layer chromatography (TLC) coupled to gas chromatography for lipid quantification. The FA content per lipid class as well as their FA composition are shown in **Figures 3B**, **4**, respectively. The following sections are subdivided into the different categories of polar and neutral lipids.

#### 3.4.1 Plastidial lipids: Galactolipids and sulfolipids

From the galactolipids, MGDG showed the most significant changes in content, while the DGDG remained constant at 1.2% w/w. The content of MGDG was highest at 29°C (1.5% w/w) and decreased to 0.9% and 0.7% at 31 and 9°C Alterations in the ratio of MGDG to DGDG are associated to variations in the degree of grana stacking in the thylakoid membranes (Jouhet, 2013; Demé et al., 2014). The decrease of MGDG at low temperatures indicates a transition of thylakoid membranes to lamellar structures similarly to high light conditions. This membrane arrangement is meant to help the repair and turnover of damaged D1 proteins from the PSII (Pribil et al., 2014).

Galactolipid contains the highest density of EPA in the cellular fraction, accompanied by a high fraction in C14:0, C16:0 and C16:1. EPA (C20:5) composition at 29°C in MGDG and DGDG was 35% and 27%, respectively, and increased to 50% and 33% at 9°C. Low temperatures reduce the fluidity of the membranes while high temperatures cause the opposite effect. As a response, cells adapt to low temperatures and high temperatures by adapting the desaturation degree of fatty acids in membranes (Los and Murata, 2004; Los et al., 2013). C16:0 decreased in both lipid classes, and C16:1 remained constant in MGDG while it increased in DGDG. Willette and coauthors reported a significant decrease in C32:1 MGDG species possibly in favour of DGDG (Willette et al., 2018). DGDG is a glycosylation product derived from MGDG. The decrease of C16:0 and C16:1 in MGDG and the increase of C16:1 in DGDG coupled with the fact that DGDG did not change with temperature implies a dynamic turnover between MGDG and DGDG. The increase of PUFAs in both MGDG and DGDG has been proven indispensable for maintaining the correct function of the plastid membranes. Interestingly, the peak of C20:5 content on a lipid basis was observed at 9°C.

Previous research showed that knocking out the elongation of C16:0 in *Nannochloropsis gaditana* resulted in a reduced content in MGDG and EPA (Dolch et al., 2017). As a consequence, photosynthesis was affected by a reduction in the electron transfer flow and an increase of non-photochemical quenching (Dolch et al., 2017). As previously mentioned, MGDG induces a membrane curvature in the thylakoid membranes. This membrane curvature, among other functions, facilitates the solubilisation of violaxanthin and related enzymes from the xanthophyll cycle and provides stability to the photosystems by keeping an adequate protein-to-lipid ratio (Dolch et al., 2017). The fatty acyl moieties are also important to this function, as shown by an increased sensitivity to cold conditions of a mutant of *Lobosphaera incisa* containing MUFAs instead of PUFAs (Zorin et al., 2017). The increase of non-photochemical quenching observed at 9°C was accompanied by a constant MGDG content coupled with an increase of C20:5 in its fatty acid composition. This might help the membrane integrity of the thylakoid, and more specifically the photosystems, by facilitating the non-photochemical dissipation of light energy and therefore preventing photodamage.

Concerning the SQDG content, it decreased from 1.2% to 0.7% w/w at suboptimal temperatures. The fatty acid composition remained relatively constant over the temperature and only the MUFA C16:1 increased from 34 to 44% from high to low temperatures at the expense of small quantities of C14:0. Our observations contrast the results from Willette and coauthors, who observed an increase at low temperatures instead of a decrease as observed in this work. Previous work in plants has shown that SQDG decreased at low temperatures due to the high content in SFA, which favours an undesired gel phase at suboptimal temperatures (Harwood, 1998). The observation of EPA (C20:5) in SQDG was not previously documented in this lipid class. This could be explained by the spillage of PC during TLC migration since they showed similar retention times at low temperatures

#### 3.4.2 Extraplastidial lipids: Phospholipids and betaine lipids

Diacylglyceryltrimethylhomo-serine (DGTS) and the phospholipids phosphatidylcholine (PC) and phosphatidylethanolamine (PE) play a central role in the desaturation and elongation of C18 fatty acids to C20:5. PE increased steadily 3-fold from 0.1% w/w at 31°C to 0.3% w/w at 9°C, while PG was constant at suboptimal and optimal temperatures, but decreased 1.5-fold at 31°C. DGTS increased significantly twofold (0.4% to 0.8% w/w) from 31°C to 9°C. As expected, PUFAs presence (and MUFAs in the case of PC) was also noticeably increased in the phospholipids PC, PG DGTS and PE (**Figure 4** and **Figure S3**). PC-C18:1 and PC-18:2 decreased at low-temperature conditions while the PC-C18:3 remained relatively constant (except at 9°C, where the PC18:3 completely disappeared). Previous studies in *Lobosphaera incisa* observed similar results and reported that the accumulation of C18:3 at low temperatures might be due to a bottleneck in the elongation step from C18:3 to PUFAs (Zorin et al., 2017). The increase of C20:5 in PC at low temperature might have been due to a possible spillage of material during analysis which could have heavily influenced the result. C20:5 in DGTS increased steadily at low temperatures at the expense of C16:0 and C16:1. In the case of PE, C20:5 increased at the expense of only C16:0, although standard deviations are considerably big to underline a trend. PG-C16:0 decreased by twofold while the C16:1 increased modestly at low temperatures.

DGTS is mainly located in the endosplasmatic reticulum (ER) and it was revealed to substitute the phospholipids PC and PE under phosphate deprivation (Mühlroth et al., 2017) or to be crucial for adaptation to low temperatures (Murakami et al., 2018). Our results also show a leading role for DGTS in regulating adaptation at decreasing temperatures. The high EPA observed in DGTS (40-60% w/w on FA basis) indicates a clear role in biosynthesis although there is still uncertainty on its functional role. Han and coworkers hypothesised a similar role in DGTS as PE in the elongation and desaturation of C20 intermediates to EPA (Han et al., 2017). In contrast, Murakami and coworkers discarded a synthesis role and suggested an end pool of EPA, competing with MGDG and DGDG in the thylakoids (Murakami et al., 2018). This behaviour was also observed at 17°C, where a decrease in MGDG was accompanied by an increase in DGTS with an increase in EPA fraction in the later one. Nevertheless, EPA fraction in MGDG and DGTS increased substantially at 9°C, emphasising a certain degree of coordination between these two lipids classes at different temperature levels. Overall, further research is needed to disentangle the role of this lipid class in the synthesis of EPA and its function under different environmental conditions.

PG is, after MGDG and DGTS, the lipid class that accumulates more C20:5. PG can also be found in the chloroplast, and therefore no distinction was done between plastidial and extraplastidial PG in this research. The composition of C20:5 was relatively constant and slightly increased at low temperatures. This was accompanied by a sharp decrease in C16:0 and a small increase in C16:1. Predominance of C16:0/C16:0 and C16:0/C6:1 in PG has been related to gel formation in plastid membranes and has been a hallmark of chilling-sensitive plants (Murata and Yamaya, 1984). Besides, the accumulation of C18:0 species was reported to facilitate phase transition in chilling conditions. In our study, we did not observe any increase in C18:0 species, which means that *N. oceanica* might be using PG species enriched with C16:1 for survival at low temperatures.

#### 3.4.3 Neutral lipids: Triacylglycerols (TAG), diacylglycerols (DAG) and free fatty acids (FFA)

Triacylglycerols (TAG) are the most abundant neutral lipid, in comparison to diacylglycerols (DAG) and free fatty acids (FFA). All neutral lipids changed in content with temperature, although the biggest differences were associated with TAG (**Figure 3B**). TAG increased 1.4-fold from 29 to 17°C and decreased 1.5-fold down at 9°C. At 31°C TAG also increased 1.4-fold, reaching a content that was similar to 17°C. The FA composition in TAG also changed with temperature and was characterised by an increase in MUFAs and PUFAs at low temperatures at the expense of a decrease in SFAs (**Figure 4**). The SFAs decreased from 60% at 31°C to 56% at 29°C, and then further to 29% at 9°C, being C16:0 the FA that showed the largest changes. C16:1 was the only MUFA that increased from 32% at 31°C to 56% at 9°C. The PUFAs C20:5 and C20:4 remained relatively constant (5% and 1%, respectively) from 31°C to 17°C and doubled at 9°C. DAG content changed with temperature in a similar fashion to TAG, with the exception of higher temperatures, where the content at 31°C did not change and remained constant at 0.15% w/w. Intracellular content of free fatty acids decreased significantly 1.5-fold from high to low temperatures, being mainly C20:4 and C20:5 the two fatty acids that decreased in content (**Figure S3**). Regarding to the FA composition in DAG, MUFAs increased at lower temperatures from 18% at 31°C to 33% at 9°C, while PUFAs and SFA did not show a clear trend. In the case of the FFA, no clear trend was observed, except that PUFAs decreased from 41% at 17°C to 18% at 9°C and SFAs increased from 37% at 17°C to 62% at 9°C.

The accumulation of FA in triacylglycerols responds to a protective cellular mechanism to safely dissipate the excess of carbon, energy and electrons under unfavourable conditions. Both low and high temperatures result in the accumulation of reducing equivalents, which can lead to the accumulation of reactive oxygen species if they are not correctly disposed. Therefore, the synthesis of TAG serves as a complementary safety mechanism to the non-photochemical quenching mechanisms abovementioned. The different FA composition in TAG at suboptimal (below 29°C) and supraoptimal conditions (above 29°C) imply that different metabolic routes occur at suboptimal and supraoptimal conditions related to the synthesis of TAG. Previous work on *Nannochloropsis salina* indicated that DAG destined for TAG synthesis mainly originated from the remodelling of lipid membranes at low temperatures (Gill et al., 2018). In that regard, PC and MGDG are well-known donors of DAG for TAG synthesis under heat stress (Mueller et al., 2017). The lower content of MGDG, instaed of PC, observed at 17°C indicates that MGDG might act as the main donor of FA for TAG synthesis. At higher temperatures, the overall increased enzymatic activity coupled to a lower content in DAG and a higher content in MGDG might explain a higher share of TAG synthesis *de novo*. The observations from this study need to be further validated with lipidomics and transcriptomics study to elucidate the metabolic routes that are activated at high and low temperatures.

### 3.5 EPA content in *Nannochloropsis oceanica* does not change at low temperatures

**Figure 5:**
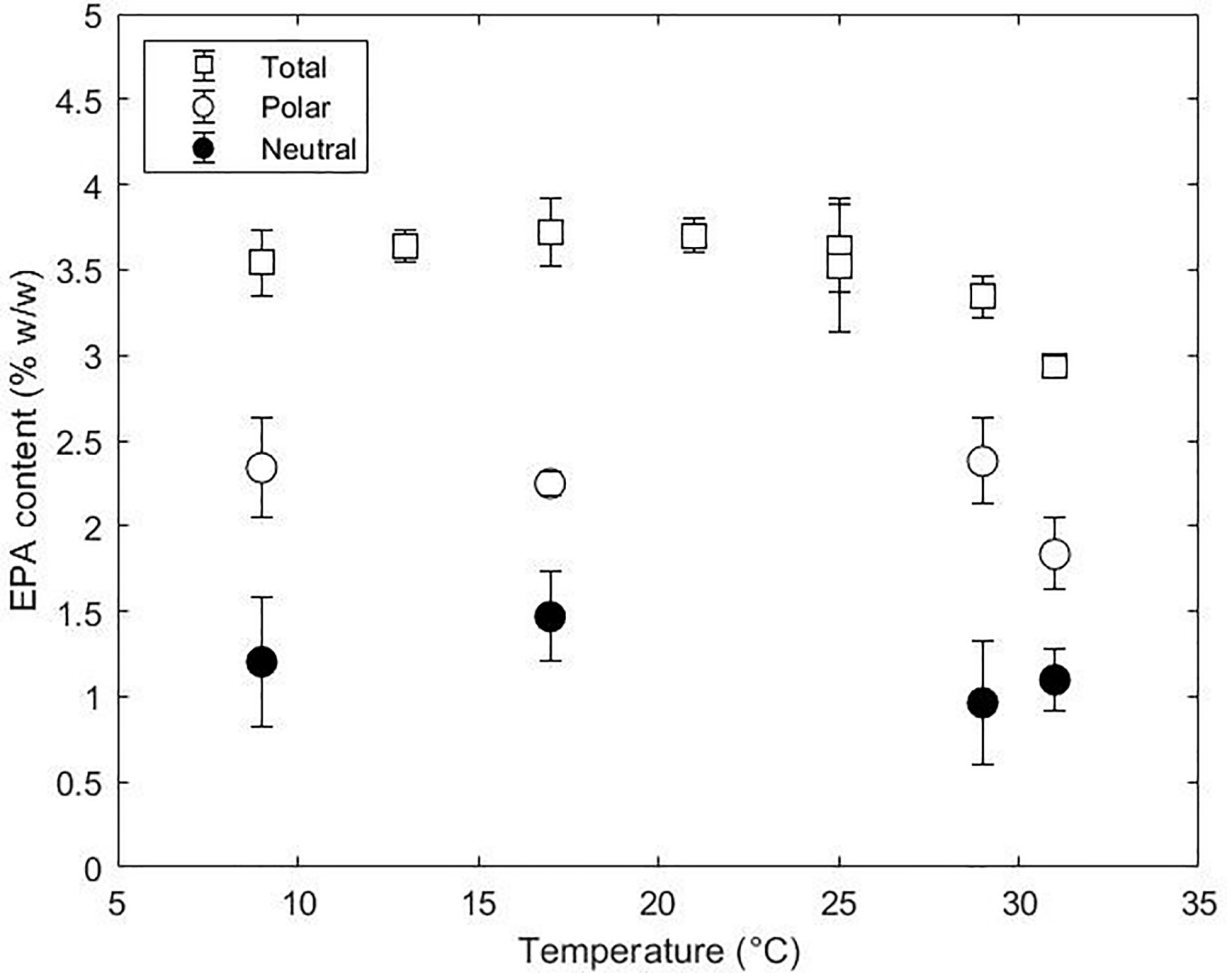
Total EPA content (%, w/w) and distribution between polar and neutral lipids at 9, 17, 29 and 31°C.

*Nannochloropsis* species are interesting from an industrial point of view since they accumulate high contents of EPA in comparison to other microalgae-producing species. Nevertheless, the economic profitability is dependent on the productivities which are currently still low for photoautotrophic systems. Lowering the temperature is regarded as a strategy to increase the EPA contents and productivities despite the growth impairment (Hoffmann et al., 2010). The total EPA content and distribution between polar and neutral lipids obtained in this research is shown in **Figure 5**. The total EPA remained constant at 3.5% w/w with decreasing temperature from 29 to 9°C and only decreased to 3.0% w/w from 29 to 31°C. The decrease in total EPA content was due to a decrease in EPA in the polar fraction (2.4 to 1.7% w/w). In contrast, EPA in the neutral fraction did not change over the range of temperatures tested. Despite the constant EPA content in the polar fraction, the content between lipid classes changed with temperature indicating a dynamic mobilisation of EPA (**Figure S4**). The increase of EPA content in DGTS, TAG and galactolipids at low temperatures indicates that these lipid classes play a relevant role in cell adaptation. Despite the changes in FA content in the polar fraction, the net content in EPA was constant and only affected at high temperatures due to a need for saturated fatty acids. The constant EPA content with the dynamics changes in lipid classes (and therefore their EPA content) indicates a tight regulation of synthesis and degradation of EPA.

Several experimental studies evaluated the effect of temperature on different species of *Nannochloropsis* (**Table 2**). Most of these studies reported an increase in EPA at slightly suboptimal temperatures. In contrast, our work did not show any changes at low temperatures. Several reasons could explain this divergence including the scale, the incident light intensity, the light pattern (continuous or circadian cycles) or the operation mode (batch or continuous) used in the experiments. Batch studies employing the same microalgae species did not result in changes in the EPA content after changing the temperature setpoint from 25 to 15°C with similar incident light intensities (Sá et al., 2020). One common feature is that all studies that reported an increase of EPA were performed at incident lights ranging from 60 to 250 µmol m^-2^ s^-1^. Our study was done at a higher incident light intensity (670 µmol m^-2^ s^-1^) which is higher than the I_ph,sat_ reported for *Nannochloropsis* species (Fisher et al., 1996). Although this needs to be confirmed with further research, the incident light intensity might influence the cellular accumulation of EPA. This might be due to the fact that a low incident light combined with low temperatures could lead to a lower redox imbalance in contrast to higher incident light intensities and low temperatures.

**Table 2:** EPA content at optimal and suboptimal conditions of different *Nannochloropsis* species at different experimental conditions (mode, scale and incident light intensity).

Species	Mode	Scale	I_ph,o_	EPA optimal	EPA suboptimal	Source
		L	*µmol m^-2^ s^-1^*	*%, w/w*	*%, w/w*	
*Nannochloropsis sp*	Batch	-	150 (L:D/12:12)	0.22 ± 0.06 (25°C)	0.35 ± 0.05 (18°C)	(Sukenik, 1991)
*Nannochloropsis salina*	Turbidostat	1	200 (CL)	2.5 ± 0.1 (26°C)	3.3 ± 0.2 (21°C) 3.5 ± 0.5 (17°C)	(Hoffmann et al., 2010)
*Nannochloropsis oculata*	Batch	60	150 (CL)	2.11 (25°C)*	2.67 (20°C)*	(Wei et al., 2015)
*Nannochloropsis salina*	Batch	0.5	250 (L:D/16:8)	3.17 ± 0.63 (25°C)	4.91 ± 0.71 (15°C) 3.00 ± 0.87 (5°C)	(Willette et al., 2018)
*Nannochloropsis oculata*	Batch	-	60 (CL)	1.56 (26°C)	2.47 (14°C) 2.45 (8°C)	(Aussant et al., 2018)
*N. oceanica*	Batch	1.8	636 (CL) 636 (L:D/16:8)	4.3 (CL, 25°C)* 4.0 (L:D, 25°C)*	5.0 (CL, 15°C)* 4.0 (L:D, 15°C)*	(Sá et al., 2020)
*N. oceanica*	Turbidostat	1.8	670 (L:D/16:8)	3.58 ± 0.47 (25°C)	3.72 ± 0.20 (17°C) 3.54 ± 0.19 (9°C)	*This work*

CL stands for continuous light and L:D stands for Ligth:Dark cycles. Asterisks indicate that values were estimated from the graphs in the original articles.

Temperature in outdoors systems is barely controlled and fluctuation can occur in the range of 10°C over the day (Béchet et al., 2010). Besides daily variations, seasonal temperatures can also influence growth and restrict microalgae cultivation to certain periods during the year depending on the thermotolerance of the species. Despite daily variations can occur in small frames of time over the day, both the EPA and the biomass productivity of the culture can be greatly affected. Biomass and EPA productivity followed a similar profile with an optimum at temperatures between 21 and 25°C (**Figure S5**). These results coincide with previous work which indicated that the optimal EPA productivity was found at temperatures optimal for growth (Chini Zittelli et al., 1999; Camacho-Rodríguez et al., 2014). However, a 10°C degree reduction from 25 to 15°C results in a 2-fold decrease in both biomass and EPA productivity. A different behaviour is observed at temperatures higher than optimal where the EPA productivity is more affected than biomass due to the reduced content in EPA. It is therefore indispensable to know in advance how temperature affects growth and product accumulations for optimal reactor operations at bigger scales even when control cannot be executed.

## 4 Conclusions

In this research, the growth, photosynthetic performance and lipidome of *Nannochloropsis oceanica* were studied in continuous cultures adapted to suboptimal, optimal and supraoptimal temperatures. Optimal growth and photosynthetic performance were found at 25-29°C. Suboptimal and supraoptimal temperatures negatively affected biomass growth and photosynthesis rates in a different extent. Growth was heavily limited at supraoptimal temperatures by the abrupt damage on photosynthetic machinery. Low temperatures triggered a profound remodelling of lipid classes and especially the polar lipid fraction and their fatty acid composition. A tipping point was found at 17°C with distinct changes in the lipidome and changes in quenching mechanisms. Diacylglyceryltrimethylhomo-serine content and desaturation level were increased at low temperatures supporting a functional role of this lipid class in adaptation. Contrary to the general belief that EPA content is increased at low temperatures, it remained constant at suboptimal temperatures and only decreased at 31°C.

## Data availability statement

The raw data supporting the conclusions of this article will be made available by the authors, without undue reservation.

## Author contributions

NF-L: Conceptualisation, Methodology, Software, Validation, Formal analysis, Investigation, Data curation, Writing – Original Draft, Writing – Review&Editing, Visualisation. LS: Conceptualisation, Methodology, Validation, Formal analysis, Investigation, Data curation, Writing – Review&Editing. RW: Conceptualisation, Supervision, Writing – Review&Editing, Project administration, Funding Acquisition. MJ Supervision, Formal analysis, Writing – Review&Editing, Visualisation. MB: Conceptualisation, Supervision, Writing – Review&Editing, Project administration, Funding Acquisition. All authors contributed to the article and approved the submitted version.
