# Supplementary material for: Growth and fatty acid distribution over lipid classes in Nannochloropsis oceanica acclimated to different temperatures
Source: Front Plant Sci. 2023 Feb 10;14:1078998. doi: 10.3389/fpls.2023.1078998 (PMC9950407; doi:10.3389/fpls.2023.1078998)
Supplement: Supplementary file 1 [file DataSheet_1.docx]

**Supplementary material**

1. SUPPLEMENTARY TABLES AND FIGURES
   1. Figures

**
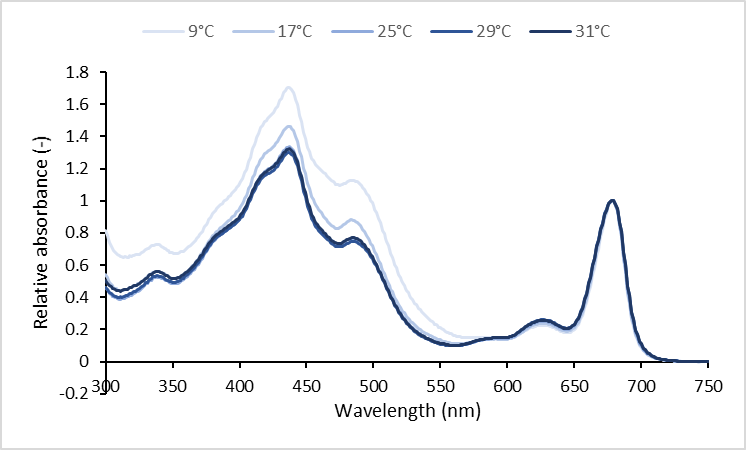
**

**Figure S1**. Absorbance spectra relative to chlorophyll absorbance peak at 680 nm at 9, 17, 25, 29 and 31 °C.

A B

**Figure S2**. (A) Ratio of the maximal specific oxygen production rate ($q_{O_{2}}^{\max}$) over the pecific respiration rate ($q_{O_{2}}^{\mathrm{dark}}$) and (B) oxygen yield on light (Y_oph_, mol_O2_ mol_ph_^-1^) at different temperatures.


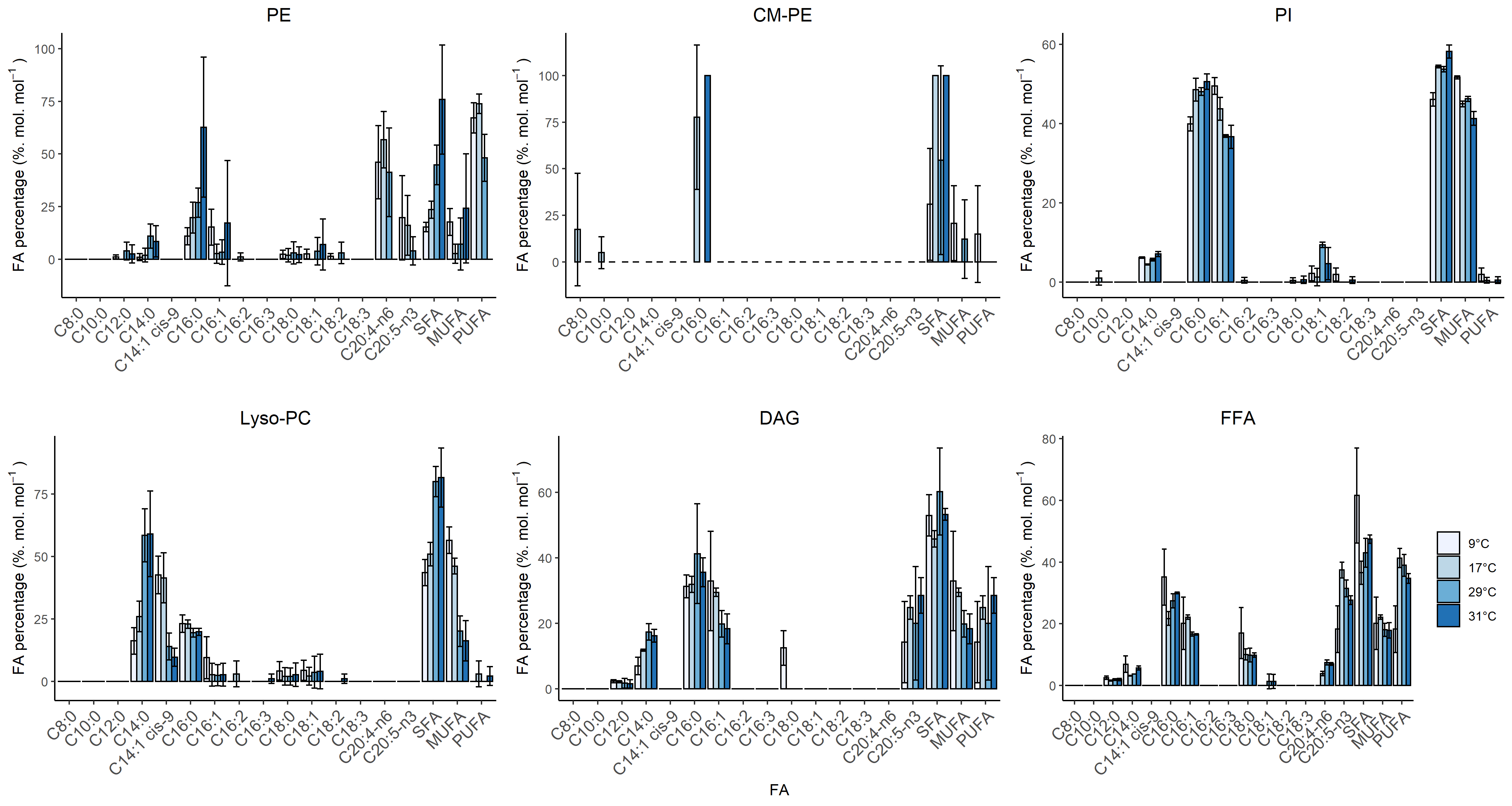


**Figure S3.** Fatty acid percentage (% mol mol^-1^), including SFA, MUFA and PUFA, of the lipid classes PE, CM-PE, PI, Lyso-PC, DAG, and FFA at 9, 17, 29 and 31 °C. Error bars indicate the standard deviation of three measurements.


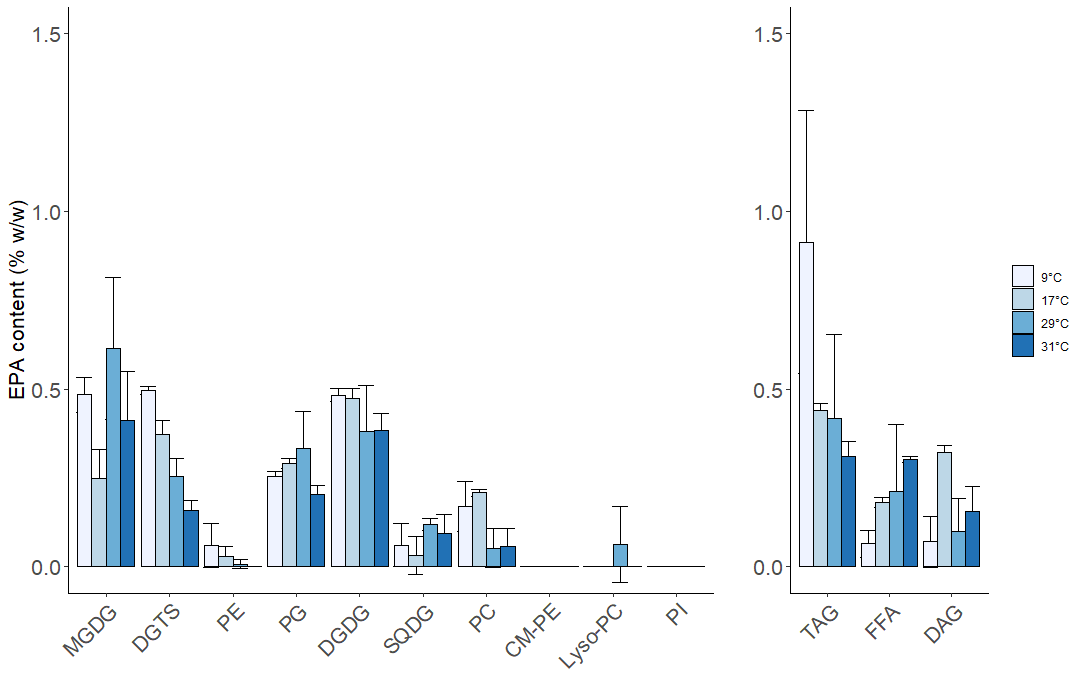


**Figure S4.** EPA content (% w/w) of polar and neutral lipid classes at 9, 17, 29 and 31 °C. Error bars represent the standard deviation of triplicate measurements.

**Figure S5.** Biomass productivity (P_X_, g L^-1^ d^-1^) and productivity of total (P_EPA_, mg L^-1^ d^-1^) and polar EPA (P_pEPA_, mg L-1 d-1) as a function of temperature (°C).

- 1. Tables

**Table S1.** FA composition (% w/w) including composition (% TFA on a lipid basis) of EPA, SFAs, MUFAs and PUFAs. Data are represented as mean ± standard deviation (n=3).

|  | **Temperature (°C)** | | | | | | |
| --- | --- | --- | --- | --- | --- | --- | --- |
|  | 9 | 13 | 17 | 21 | 25 | 29 | 31 |
|  |  |  |  |  |  |  |  |
| *C8:0* | 0.01 ± 0.01 | 0.0 ± 0.0 | 0.0 ± 0.0 | 0.01 ± 0.01 | 0.01 ± 0.02 | 0.01 ± 0.02 | 0.01 ± 0.01 |
| *C10:0* | 0.01 ± 0.01 | 0.0 ± 0.01 | 0.0 ± 0.0 | 0.02 ± 0.0 | 0.02 ± 0.01 | 0.23 ± 0.36 | 0.02 ± 0.0 |
| *C12:0* | 0.07 ± 0.02 | 0.05 ± 0.0 | 0.07 ± 0.0 | 0.07 ± 0.01 | 0.06 ± 0.01 | 0.09 ± 0.0 | 0.20 ± 0.13 |
| *C14:0* | 0.74 ± 0.26 | 0.82 ± 0.02 | 1.02 ± 0.03 | 0.91 ± 0.07 | 0.93 ± 0.12 | 1.40 ± 0.16 | 1.56 ± 0.06 |
| *C14:1 cis 9* | 0.13 ± 0.08 | 0.07 ± 0.02 | 0.05 ± 0.0 | 0.04 ± 0.0 | 0.03 ± 0.02 | 0.03 ± 0.0 | 0.03 ± 0.05 |
| *C16:0* | 2.73 ± 0.12 | 4.58 ± 0.13 | 6.34 ± 0.24 | 6.22 ± 0.63 | 5.50 ± 0.68 | 6.17 ± 0.11 | 6.98 ± 0.23 |
| *C16:1* | 3.27 ± 0.1 | 4.24 ± 0.12 | 4.58 ± 0.18 | 3.55 ± 0.26 | 2.89 ± 0.33 | 2.71 ± 0.05 | 2.74 ± 0.13 |
| *C16:2* | 0.1 ± 0.0 | 0.17 ± 0.01 | 0.15 ± 0.01 | 0.08 ± 0.07 | 0.07 ± 0.07 | 0.04 ± 0.08 | 0.04 ± 0.05 |
| *C18:0* | 0.61 ± 0.09 | 0.34 ± 0.07 | 0.32 ± 0.04 | 0.31 ± 0.05 | 0.26 ± 0.04 | 0.44 ± 0.01 | 0.59 ± 0.06 |
| *C18:1* | 0.42 ± 0.06 | 0.48 ± 0.05 | 0.53 ± 0.03 | 0.77 ± 0.14 | 0.61 ± 0.07 | 0.64 ± 0.01 | 0.60 ± 0.06 |
| *C18:2* | 0.19 ± 0.02 | 0.25 ± 0.03 | 0.19 ± 0.01 | 0.28 ± 0.04 | 0.29 ± 0.03 | 0.38 ± 0.01 | 0.38 ± 0.01 |
| *C18:3* | 0.05 ± 0.009 | 0.0 ± 0.0 | 0.15 ± 0.13 | 0.27 ± 0.02 | 0.20 ± 0.17 | 0.08 ± 0.15 | 0.17 ± 0.15 |
| *C20:4* | 0.65 ± 0.09 | 0.74 ± 0.04 | 0.72 ± 0.03 | 0.74 ± 0.05 | 0.64 ± 0.11 | 0.59 ± 0.03 | 0.59 ± 0.04 |
| *C20:5* | 3.54 ± 0.19 | 3.64 ± 0.09 | 3.72 ± 0.2 | 3.70 ± 0.10 | 3.58 ± 0.47 | 3.34 ± 0.12 | 2.93 ± 0.06 |
| *SFA* | 4.16 ± 0.3 | 5.79 ± 0.15 | 7.74 ± 0.25 | 7.52 ± 0.64 | 6.77 ± 0.69 | 8.34 ± 0.41 | 9.36 ± 0.28 |
| *MUFA* | 3.82 ± 0.15 | 4.78 ± 0.13 | 5.16 ± 0.18 | 4.35 ± 0.29 | 3.52 ± 0.34 | 3.38 ± 0.05 | 3.36 ± 0.15 |
| *PUFA* | 4.49 ± 0.23 | 4.81 ± 0.11 | 4.78 ± 0.24 | 4.80 ± 0.14 | 4.56 ± 0.53 | 4.36 ± 0.21 | 3.94 ± 0.17 |
| *TFA* | 12.47 ± 0.41 | 15.38 ± 0.22 | 17.68 ± 0.39 | 16.68 ± 0.72 | 14.86 ± 0.93 | 16.08 ± 0.46 | 16.66 ± 0.36 |
| *C20:5 (% TFA)* | 28.43 ± 1.81 | 23.70 ± 0.69 | 21.02 ± 1.22 | 22.19 ± 1.12 | 24.07 ± 3.53 | 20.79 ± 0.96 | 17.60 ± 0.53 |
| *SFA (% TFA)* | 33.41 ± 2.65 | 37.64 ± 1.10 | 43.79 ± 1.70 | 45.11 ± 4.29 | 45.59 ± 5.47 | 51.86 ± 2.97 | 56.17 ± 2.05 |
| *MUFA (%TFA)* | 30.61 ± 1.54 | 31.09 ± 0.98 | 29.16 ± 1.22 | 26.10 ± 2.07 | 23.68 ± 2.73 | 21.01 ± 0.69 | 20.19 ± 1.00 |
| *PUFA (%TFA)* | 35.98 ± 2.21 | 31.27 ± 0.82 | 27.05 ± 1.49 | 28.80 ± 1.49 | 30.72 ± 4.04 | 27.14 ± 1.51 | 23.64 ± 1.16 |
|  |  |  |  |  |  |  |  |
